# Cancer survivorship in malignant melanoma

**DOI:** 10.1111/ddg.70486x

**Published:** 2026-09-08

**Authors:** Markus Reitmajer, Susanne Dugas‐Breit, Chiara L. Blomen, Elisabeth Livingstone, Max Schlaak, Markus V. Heppt, Michael M. Sachse, Madlen Hörold, Friedegund Meier, Sebastian Haferkamp, Christian Apfelbacher, Georgia Schilling, Andrea von den Berg, Frank Meiss, Lisa Zimmer, Andrea Forschner

**Affiliations:** ^1^ Department of Dermatology University Hospital Tuebingen Tuebingen Germany; ^2^ Department of Medical Oncology National Center for Tumor Diseases (NCT) Heidelberg University Hospital Heidelberg Germany; ^3^ Department of Dermatology and Venereology University Medical Center Hamburg‐Eppendorf (UKE) Hamburg Germany Fleur Hiege Center for Skin Cancer Research University Medical Center Hamburg‐Eppendorf (UKE) Hamburg Germany; ^4^ Department of Dermatology University Hospital Essen Essen Germany; ^5^ Department of Dermatology Venereology and Allergology Charité‐Universitätsmedizin Berlin corporate member of Freie Universität Berlin and Humboldt‐Universität zu Berlin Berlin Germany; ^6^ Department of Dermatology Uniklinikum Erlangen Friedrich‐Alexander‐University Erlangen‐Nürnberg (FAU) Erlangen Germany; ^7^ Comprehensive Cancer Center Erlangen‐European Metropolitan Area of Nuremberg (CCC ER‐EMN) Erlangen Germany Bavarian Cancer Research Center (BZKF) Uniklinikum Erlangen Friedrich‐Alexander‐University Erlangen‐Nürnberg (FAU) Erlangen Germany; ^8^ Dermpath München Laboratory for Dermatopathology Oral Pathology and Molecular Pathology Munich Germany; ^9^ Department of Dermatology Allergology and Phlebology Bremerhaven Reinkenheide Bremerhaven Germany; ^10^ Institute of Social Medicine and Health Systems Research Medical Faculty Otto von Guericke University Magdeburg Magdeburg Germany; ^11^ Skin Cancer Center at the University Cancer Centre Dresden and National Center for Tumor Diseases Dresden Germany; ^12^ Department of Dermatology Faculty of Medicine and University Hospital Carl Gustav Carus Technische Universität Dresden Dresden Germany; ^13^ Department of Dermatology University Hospital Regensburg Regensburg Germany; ^14^ Department of Dermatology University Hospital and Faculty of Medicine Martin Luther University Halle‐Wittenberg Halle (Saale) Germany; ^15^ Department of Oncological Rehabilitation Asklepios Nordseeklinik Westerland, Germany; ^16^ Department of Oncological Rehabilitation Asklepios Klinik Triberg Triberg Germany; ^17^ Department of Dermatology Medical Center – University of Freiburg Faculty of Medicine University of Freiburg Freiburg Germany

**Keywords:** follow‐up, health‐related quality of life (HRQoL), immune checkpoint inhibitor (ICI), immune‐related adverse events (irAEs), melanoma, quality of life (QoL), survivorship, surveillance

## Abstract

Robust long‐term survival data have transformed the prognosis of metastatic melanoma. For combination therapy with immune checkpoint inhibitors (ICIs), 10‐year melanoma‐specific survival rates now exceed 50 %, and durable disease control is achievable in a substantial proportion of patients, including those with brain metastases.

With increasing long‐term survival, chronic and late‐onset treatment‐related toxicities are becoming increasingly relevant. In particular, chronic immune‐related adverse events (irAEs) may persist after discontinuation of ICI therapy or emerge with delayed onset. These toxicities can significantly impair long‐term quality of life and often require ongoing management; endocrine toxicities, for example, may necessitate lifelong hormone replacement therapy.

In addition to physical sequelae, survivors frequently experience psychosocial and financial burdens, difficulties returning to work, and concerns related to fertility and family planning. These multidimensional challenges highlight the growing importance of survivorship care in patients treated with ICIs.

Systematic assessment of physical and psychosocial burden as well as health‐related quality of life, especially through patient‐reported outcomes, can support clinical decision‐making and individualized follow‐up strategies. Integrating these assessments into structured, multidisciplinary survivorship care models is essential to address the complex long‐term needs of melanoma survivors and to optimize outcomes beyond disease control.

## DEFINITION AND TERMINOLOGY OF LONG‐TERM SURVIVAL (*SURVIVORSHIP*)

The number of patients surviving a cancer disease is steadily increasing.^1^ The causes are manifold and multifactorial. Apart from increasing life expectancy and the demographic change, improved screening programs, optimized diagnostic workup, and innovative treatment options contribute significantly to improved survival after a diagnosis of cancer. Based on current estimates, almost 1.5 million people live with and after melanoma in the USA,^1^ while in Germany more than 300,000 people live with and after this disease.^2^

Two thirds of these melanoma survivors in Germany are long‐term survivors who received their initial diagnosis at least five years previously. Almost half of them are still of working age or younger than 65 years old.^2^ A comparable situation is apparent in the USA, where more than 70 % of melanoma survivors are long‐term survivors, 40 % are of working age, and 12 % are younger than 50 years old.^1^

### *Cancer survivor* and survivorship

**FIGURE 1:**
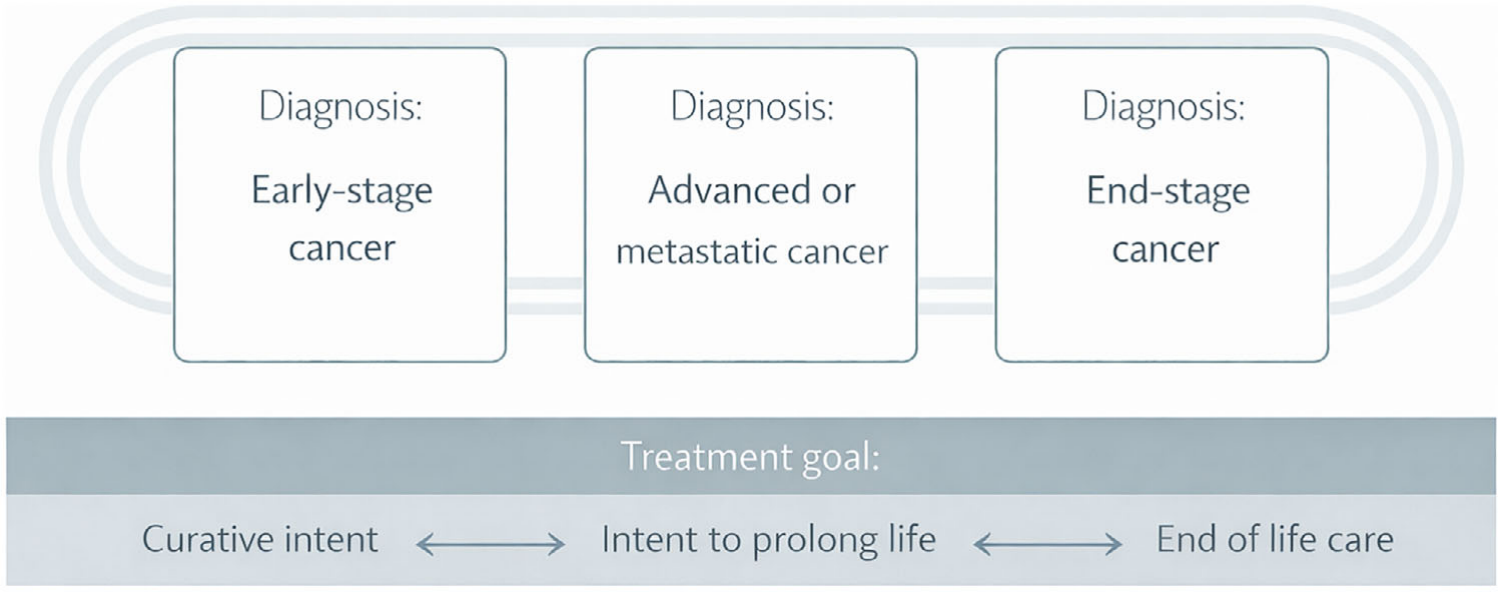
Goals of care across stages of cancer. The figure illustrates the continuum of cancer care from early‐stage disease through advanced or metastatic cancer to end‐stage disease. It highlights the evolving goals of care, shifting from curative intent to life‐prolonging treatment strategies and ultimately to palliative and end‐of‐life care. Transitions between phases are shown as dynamic and may vary across individual patients. The figure was generated using abacus.ai software (Abacus.AI, San Francisco, CA, USA).

*Cancer survivor* refers to any person from the time of cancer diagnosis until the end of life – irrespective of disease stage, prognosis, or disease course.^3^ Accordingly, *cancer survivors* constitute a heterogeneous group of patients in different phases of their cancer disease and therefore have different requirements and an individual support needs (Figure 1).^4^ In the context of melanoma, this means that melanoma survivors include both patients with an initial diagnosis of melanoma after complete tumor excision and patients with advanced disease receiving, for example, systemic therapy. The term long‐term survivors usually refers to those patients who received their initial cancer diagnosis at least 5 years previously.^5^

### Long‐term survival data in melanoma

For melanoma, robust data on 10‐year survival after immune checkpoint inhibitor (ICI) therapy from two landmark phase 3 studies are available.^6^, ^7^ In the Checkmate 067 study, therapy‐naïve patients with metastatic melanoma showed an overall 10‐year survival rate of 43 % under combined therapy with ipilimumab and nivolumab and of 37 % with nivolumab monotherapy.^6^ The melanoma‐specific 10‐year survival was 52 % with combined ICI therapy and 44 % with nivolumab monotherapy. Notably, patients experiencing no progression three years after initiation of therapy with ipilimumab and nivolumab or nivolumab monotherapy showed a melanoma‐specific survival of ≥ 96 %. The depth of response proved to be another important predictor of long‐term survival. Patients with a tumor reduction of ≥ 80 % had a melanoma‐specific 10‐year survival of ≥ 87 %.^6^

Similar results were obtained in the Keynote‐006 study.^7^ Patients treated with the anti‐PD1 antibody pembrolizumab showed an overall 10‐year survival of 34 % and a melanoma‐specific 10‐year survival of 45 %. Analogous to the Checkmate 067 study, patients with complete remission (CR) had the most favorable melanoma‐specific 8‐year survival with 88 % compared to 68 % in patients with partial tumor response (PR) and 36 % in patients with stable disease (SD).^7^ Another important aspect of the Keynote‐006 study is the finding that patients do not need continued ICI treatment to achieve long‐term survival. Of those patients who had discontinued pembrolizumab after two years due to CR/PR/SD, 65 % were free of progression after eight years and more than 80 % were still alive at that time.^7^

In the Checkmate 067 study, therapy‐naive patients with metastatic melanoma showed an overall 10‐year survival of 43 % under combined therapy with ipilimumab and nivolumab, the melanoma‐specific 10‐year survival was 52 %. With such a long follow‐up, the melanoma‐specific survival has a particularly high significance.^6^

Similarly, long‐lasting remissions are achieved with anti‐PD‐1 treatment as monotherapy.^6^ The melanoma‐specific 10‐year survival with nivolumab and pembrolizumab monotherapy was 44 % and 45 %, respectively.^6^, ^7^

### Long‐term data in case of brain metastasis: The ABC study

Brain metastases occur in 30–40 % of patients with melanoma and present a particular clinical challenge. Long‐term data are now also available for this particular group of patients. In the Australian randomized multicenter phase 2 study “Anti‐PD‐1 Brain Collaboration” (ABC), ICI‐naïve patients with asymptomatic brain metastases showed an overall 7‐year survival of 48 % under combined ICI therapy with ipilimumab/nivolumab compared to 26 % with nivolumab monotherapy.^8^ The intracranial response rate was 51 % for combination therapy versus 20 % for nivolumab monotherapy.^8^ The data underscore impressively that long‐term disease control can be achieved with ipilimumab/nivolumab in a substantial proportion of patients with brain metastases.

## CHRONIC AND LATE‐ONSET TOXICITIES UNDER IMMUNOTHERAPY AND TARGETED THERAPY IN MELANOMA

### Chronic and late‐onset toxicities under immunotherapy

With the increasing number of long‐term survivors among melanoma patients, persistent and late‐onset treatment‐related toxicities become increasingly relevant. Although acute immune‐related adverse events (irAEs) under ICI treatment are well characterized, their chronification has been underestimated for a long time. Recent prospective and retrospective analyses show, however, that a relevant proportion of treated patients suffer from persistent functional impairments that may permanently impair their quality of life (QoL). A consistent terminology is essential for clinical routine, comparability across studies, and systematic assessment of late‐onset toxicities. In an international consensus paper, the *Society for Immunotherapy of Cancer* (SITC) defined “chronic irAEs” as adverse events persisting beyond three months after discontinuation of ICI therapy. In addition, “chronic active” and “chronic inactive” irAEs are distinguished according to the presence of persistent inflammation and/or the requirement for immunosuppressive treatment.^9^ Adverse events emerging within six months after therapy discontinuation are considered “delayed” or “*late‐onset* irAEs”. For adverse events emerging after treatment discontinuation in particular, establishing the causal link to ICIs is difficult, especially if patients have received follow‐on therapies.

Current prospective and retrospective analyses show, however, that a relevant proportion of treated patients suffer from persistent functional impairments that may permanently impair the quality of life (QoL).

The most detailed data available to date on chronification of irAEs in melanoma come from a cohort of high‐risk patients receiving adjuvant anti‐PD‐1 therapy. In this retrospective study, 43 % of 387 patients treated with adjuvant anti‐PD‐1 therapy developed at least one chronic irAE.^10^ In the extended follow‐up of this cohort,^11^ symptoms persisted for more than 1.5 years after therapy discontinuation in 29 % of affected patients, and 45 % of the chronic irAEs remained symptomatic. Particularly common were chronification of adrenal insufficiency, hypothyroidism and thyroiditis, neuropathy, and nephritis. Although fatigue was also often reported by patients, the authors excluded fatigue from the statistics because the causal attribution to ICIs is difficult. In a recent German study among long‐term melanoma survivors (≥ 5 years after initial diagnosis of stage IV disease), more than 40 % of patients reported chronic adverse events.^12^

**FIGURE 2:**
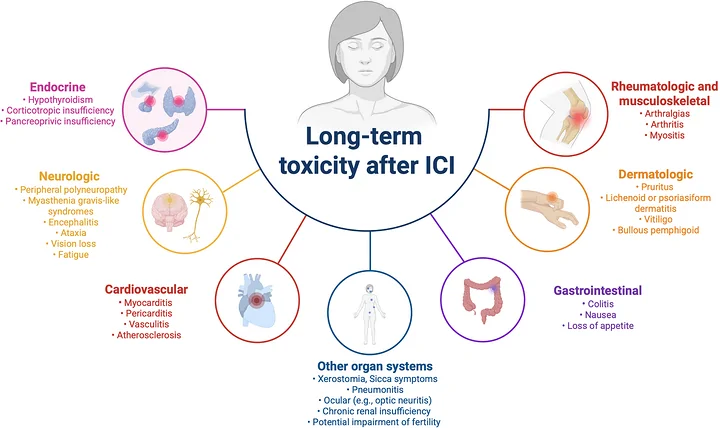
Long‐term toxicities following immune checkpoint inhibitor (ICI) therapy. The figure illustrates the most common long‐term toxicities following treatment with immune checkpoint inhibitors (ICIs). Typical clinical manifestations are shown, stratified by the affected organ systems. The figure was generated using BioRender software (BioRender.com, Toronto, Canada).

Systematic reviews confirm that chronic irAEs have a clinically relevant prevalence across entities and are likely underestimated in many studies.^13^, ^14^ The mechanisms of chronic irAEs are currently not fully understood. Proposed mechanisms include irreversible organ damage during the acute phase (especially for endocrine irAEs), persistent inflammation (immune‐related arthritis), and general mechanisms underlying irAE development (cross‐reactivity between tumor antigens and autoantigens, environmental factors, microbiome, smoldering autoimmunity, tissue‐associated immune cells, and genetic factors).^14^ The organ‐related chronic and late‐onset toxicities most relevant to clinical routine, typical patterns of persistence, monitoring, and management are summarized in Table 1 and Figure 2.^10^, ^11^, ^13^, ^14^, ^15^, ^16^, ^17^, ^18^

**TABLE 1:** Organ‐specific chronic immune‐related adverse events (irAEs) – Frequency – Persistence – Management. Frequency estimates vary depending on the study population and the definition of chronic irAEs.

Organ system	Typical chronic/late‐onset toxicities	Frequency/examples (melanoma‐specific, if available)	Pattern of persistence	Monitoring during follow‐up	Management (oriented towards survivorship)	Key references
**Endocrine (ICI)**	Hypothyroidism/thyroiditis, hypophysitis, adrenal insufficiency, (less common) diabetes	Hypothyroidism/thyroiditis chronic 14 % (of these, 85.7 % persisting > 1.5 years); hypophysitis/adrenal insufficiency completely persistent in cohort	Mostly **irreversible** (chronic inactive)	TSH/fT4, cortisol/ACTH, electrolytes, HbA1c/glucose; patient‐reported symptoms	Permanent hormone replacement, emergency pass/stress dose training, coordinated endocrinology	^11^, ^14^, ^18^
**Rheumatologic (ICI)**	Chronic arthritis/arthralgias, myositis overlap, PMR‐like	Arthritis/arthralgias chronic 5.7 %; 41 % persisting > 1.5 years	Often **recurrent/ steroid‐dependent**	Function/pain, inflammatory markers, imaging, if appropriate	Steroid‐sparing (DMARDs/biologics), physiotherapy/pain medicine	^11^, ^14^, ^18^
**Gastrointestinal (ICI)**	Chronic colitis/enteritis, microscopic colitis, malabsorption	In GI‐irAE cohort 8 % with inflammation ≥ 6 months after ICI; in adjuvant cohort, colitis rarely chronic	Months to years; sometimes **refractory to steroids**	History of bowel movement, calprotectin, endoscopy in case of persistence/recurrence	Escalation: steroids → vedolizumab/infliximab and others; nutrition/supportive; rarely surgery	^11^, ^15^
**Neurologic (ICI)**	Neuropathies, myositis/MG overlap, encephalitis/myelitis, autonomous disorders	Rare (< 5 %); in n‐irAE cohort: 57 % chronic (non‐fulminant), of these, ∼52 % chronic active	High rate of **residual deficits**; in part, chronic active	Neurological function scores, need for rehab; screening for myocarditis in myositis/MG	Early high‐dose steroids, IVIG/PLEX, rituximab, if appropriate; rehabilitation; interdisciplinary	^16^
**Dermatologic (ICI)**	Chronic pruritus/dermatitis, lichenoid/psoriasiform, BP‐like; vitiligo	Frequent (10–20 %); dermatitis/pruritus chronic 6.6 % (22.1 % persisting > 1.5 years); chronic cutaneous irAE median duration ∼629 d, persistence 54 % after 18 months	Often persistent; BP‐like particularly protracted	Dermatological follow‐ups, PRO assessment pruritus/sleep	Topical/systemic; steroid‐sparing (e. g. biologics) in case of selected patterns	^11^, ^17^
**Cardiovascular (ICI)**	Myocarditis (incl. subclinical/”smoldering”), pericarditis, vasculitis, arrhythmias; potential atherosclerotic events/plaque progression	Chronic cardiac irAEs overall rare (in reviews approx. 2 % of non‐endocrine chronic irAEs); in adjuvant anti‐PD‐1 cohort chronic myocarditis 0.3 % (persistent)	Long‐term course following myocarditis/pericarditis not well defined (risk of persistent inflammatory cardiomyopathy or later LV dysfunction unclear); potentially underestimated risk of atherosclerotic events	Low‐threshold cardio‐oncology; in case of symptoms/abnormal biomarkers: ECG, troponin/BNP, echo, cardio‐MRT, if appropriate; in addition, strict CV risk profile analysis (blood pressure, lipids, diabetes, smoking), potentially follow‐up dependent on risk constellation	Chronic residuals: HF therapy/arrhythmia management; in case of potential atherosclerosis: intensive risk factor management (statin/antihypertensives/diab control), exercise/rehab	^10^, ^13^, ^18^
**Salivary glands/oral (ICI)**	Xerostomia/Sicca (Sjögren‐like)	Xerostomia chronic 2.3 %; 88.9 % persistent	Mostly prolonged, often chronic inactive	Oral/ocular symptoms, history of infections/caries; dental controls	Substitution/stimulation of saliva, fluoridation; sialogogues (pilocarpine/cevimeline), if appropriate; dentistry/potentially rheumatology	^10^
**Pulmonary (ICI)**	Pneumonitis, persistent radiologic changes	Pneumonitis chronic 2.5 % (44.4 % persisting > 1.5 years)	Variable; recurrence possible	Clinical appearance, low‐dose CT, pulmonary function testing, bronchoalveolar lavage	Steroids; infections/tumor progression as differential diagnosis	^10^, ^11^
**Renal (ICI)**	Nephritis, CKD	Nephritis/nephrotic syndrome chronic 1.3 % (50 % persisting > 1.5 years)	In part persistent	Creatinine/eGFR, urinalysis, microprotein analysis	Steroids/immunosuppression; nephrology	^10^, ^11^
**Ocular (ICI)**	Uveitis, optic neuritis, retinal vasculitis	Ocular chronic irAEs 1.3 % (57.1 % persisting > 1.5 years)	Variable	Ophthalmologic symptom‐related/in case of relevant history	Topical/systemic; ophthalmology	^16^, ^18^

### Late‐onset toxicity under targeted therapy

BRAF and MEK inhibitors may also cause chronic adverse events, but these are significantly less common and usually not irreversible. In a large retrospective multicenter adjuvant cohort (n = 598; stage III, BRAF V600‐mutated), persistent adverse events (> 3 months after therapy discontinuation) were reported in 3 % of patients on dabrafenib/trametinib treatment versus 26 % of those on anti‐PD‐1 treatment. While arthralgias and cardiovascular effects (for example, left ventricular dysfunction on treatment with MEK inhibitors) may persistently impair QoL, they usually resolve after discontinuation of the targeted therapy (TT). Similarly, retinal edemas (MEK‐associated retinopathies) are usually transient, whereas persistent ocular adverse events, such as retinal vein occlusion, are very rare events. *Patient‐reported outcome* data from adjuvant treatment show that chronic fatigue may also occur during dabrafenib/trametinib treatment.^19^

### Financial toxicity, occupational reintegration and performance

Chronic and late‐onset toxicities often result in long‐term consequential costs (hormone replacement, repeated specialist consultations, biologics or other immunosuppressants, rehabilitation). In addition, toxicity‐related work absence may further aggravate the financial burden. Chronic active irAEs with long‐term immunosuppression or need for biologics are particularly resource‐intensive.

Occupational reintegration is not primarily limited by rare organ damage but rather by persistent symptoms, including fatigue, pain, GI‐related problems, and dermatologic pruritus. In an Australian study, 50 % of the patients reported that their working hours and income remained reduced one year after discontinuation of adjuvant ICI/TT therapy; one third reported financial toxicity.^20^ In German‐speaking countries, similar issues are gaining in importance. Accordingly, the German S3 guideline on melanoma explicitly emphasizes the the long term relevance of occupational reintegration and social medical rehabilitation.^21^ Comparable data from Germany have not yet been published (as of 05/2026).

## FERTILITY, FAMILY PLANNING, AND PREGNANCY

Malignant melanoma is one of the most common cancers in young adults of childbearing age.^22^, ^23^ In view of the long‐term survival rates achieved to date, family planning and the associated preservation of fertility are increasingly moving into the focus of oncological care.

### Impact of systemic therapies in melanoma on fertility

For the majority of patients, the loss of fertility is a major concern prior to initiation of cancer therapy and may be as stressful as the cancer diagnosis itself.^24^ Robust data on fertility after ICI or BRAF/MEK therapy are limited and the actual impact on fertility is still unclear.^23^, ^26^ Based on available summaries of product characteristics, impairment of fertility cannot be excluded.^25^ Reduced male fertility during and after TT is possible. Moreover, it must be kept in mind that due to potential fetal risks and limited evidence, strict contraception during systemic therapy is recommended; this applies to TT, in particular.

Similarly, few data are available on potential gonadal toxicity of ICIs. In a retrospective analysis of an adverse event database of the US Food and Drug Administration (FDA), 133,512 patients receiving ICI therapy were analyzed. Adverse events related to the reproductive system were identified in 0.43 %. The rate of adverse events was higher in females than in males (0.57 % vs. 0.39 %, p < 0.001). In females, vaginal bleeding and 34 cases of fistula formation in the genital tract were described. In males, changes in spermatogenesis were reported most often, especially after dual ICI therapy with anti‐PD‐1 plus anti‐CTLA‐4 antibodies. Given the low patient number (n = 6), the significance of these data is, however, limited.^26^

Fertility may also be affected indirectly. In particular, endocrine irAEs, such as hypophysitis, thyroiditis, or primary gonadal insufficiency, may result in hormonal abnormalities with adverse effects on reproductive capacity. Usually, these hormonal changes can be treated by replacement therapy in the context of endocrinological care.

### Fertility consultation before initiating medical therapy

International guidelines and expert recommendations emphasize the importance of early, structured fertility consultation for all patients of reproductive age prior to initiation of systemic oncologic therapy.^27^, ^28^ This consultation should be conducted individually and include aspects such as the desire to have children, prognosis, therapy‐related risks to fertility, necessary measures of contraception, and available options for preservation of fertility. Referral to specialized centers for reproductive medicine in a timely manner is recommended. Moreover, psychosocial support should be offered and the consultation should be documented.

### Options for preservation of fertility

According to the AWMF S2k guideline on fertility preservation for patients with malignant disease, cryopreservation of fertilized or unfertilized oocytes and ovarian tissue is recommended for females; moreover, use of GnRH agonists may be considered in selected cases.^29^ Oocyte cryopreservation requires ovarian stimulation for approximately two weeks with subsequent collection of oocytes, while ovarian tissue can be collected in the short term and retransplanted at a later time. For males, cryopreservation of ejaculate is primarily recommended; cryopreservation of testicular tissue may be an option in individual cases.^22^, ^26^, ^29^

### Pregnancy management

Although only few clinical data are available concerning BRAF and MEK inhibitors, animal experiments have indicated embryotoxic, fetotoxic, and teratogenic effects. The effect of TT and ICI therapies during pregnancy is largely unclear. In a retrospective study, 49 women receiving ICI and experiencing potential effects on reproductive organs were identified. In 45 % of the cases, the patients had received nivolumab as monotherapy. In this cohort, there were nine conceptions, two during ongoing therapy with one miscarriage and seven pregnancies after termination of therapy with two spontaneous abortions and one elective abortion.^30^

Given the potential risks to the embryo/fetus, reliable contraception is required during and, depending on approval, 4–5 months after systemic melanoma therapy. Furthermore, the efficacy of oral or systemic contraceptives may be compromised, and additional contraceptive measures should be used.^22^ In the adjuvant setting, no systemic therapy should be initiated during pregnancy. If pregnancy is detected during ongoing adjuvant treatment, discontinuation of the adjuvant systemic therapy is usually recommended. In advanced or metastatic disease, systemic therapy may be performed during pregnancy in exceptional cases and after strict individual risk‐benefit assessment. Such decisions should be made in specialized tumor boards in an interdisciplinary manner. Following completion of cancer therapy, pregnancy is possible in principle and not generally contraindicated. However, there may be an increased risk of pregnancy and birth complications, especially if there is a short interval between the end of therapy and conception. Tight gynecological and oncological care is, therefore, recommended. Moreover, there is an increased risk of recurrence after conception, while potential restrictions with respect to diagnostics and therapy apply. From an oncological perspective, consultation concerning the individual recurrence risk and the optimal time of pregnancy is, therefore, recommended.^31^, ^32^

## QUALITY OF LIFE (QOL) AS CENTRAL GOAL OF CARE IN MELANOMA PATIENTS

The QoL is a central goal of oncologic care, not only in the context of survival but also as an integral component of holistic patient care. It goes beyond purely medical endpoints and includes major physical, psychological, and social dimensions as well as the health‐related QoL (HRQoL).^33^ While chronic treatment‐related toxicities and late‐onset sequelae have been addressed in the previous section, this chapter deliberately adopts the subjective perspective of affected patients. HRQoL comprises physical as well as psychological, social, and functional aspects that are crucial for the everyday life of those affected.^34^

The QoL of melanoma survivors comprises physical, psychological, social, and functional dimensions.

Systematic assessment of QoL is highly relevant for clinical decision‐making, given that it enables a differentiated assessment of therapeutic benefit and potential treatment‐associated burden. This applies to melanoma patients, in particular, given that surgical and systemic treatments may cause late‐onset sequelae even in the survivorship phase.^12^, ^35^

Systematic assessment of QoL is essential for clinical decisions, patient consultation, and planning of follow‐up.

### Assessment of quality of life (QoL): Instruments and methods

The QoL of melanoma survivors can be assessed by various patient‐reported outcome (PRO) instruments. In principle, general (generic) questionnaires (instruments) that can be used irrespective of the disease (Table 2)^36^, ^37^, ^38^ are distinguished from HRQoL‐specific instruments depicting specifically tumor‐associated burden and therapy‐related effects (Table 3).^39^, ^40^, ^41^, ^42^ For clinical routine and scientific studies, a combination of both approaches is often advisable to cover both comparability with the general population and disease‐specific aspects. Usually, a generic instrument is used first to enable comparability between various diseases and health economic evaluations. This is complemented by an oncology‐specific HRQoL instrument and the PRO‐CTCAE, if appropriate, to specifically capture treatment‐related late‐onset sequelae. This approach allows for a valid and clinically relevant depiction of both general and melanoma‐specific effects on QoL.

**TABLE 2:** General (generic) Instruments for Assessing Quality of Life. These tools assess health‐related QoL independently of a specific disease and enable comparisons with other patient populations or the general population.

Instrument	Description
**SF‐12 / VR‐12** (*Short Form / Veterans Rand Health Survey*)	Comparable questionnaires with twelve items each assessing physical and psychological health dimensions (including physical function, vitality, social function, psychological well‐being) (subject to license and license‐free, respectively).^36^
**EQ‐5D‐3L / EQ‐5D‐5L** (EuroQol)	A short questionnaire with five core areas (mobility, self‐sufficiency, everyday activities, pain/discomfort, anxiety/depression) and a visual analog scale enabling the calculation of so‐called utility scores, that is, summarizing parameters of the patient's health status (0 = death, 1 = full health) that allow for quantification and comparison of changes in quality of life (subject to approval, usually free of charge for academic, non‐commercial use).^37^
**WHO‐5 Well‐Being Index**	Very short generic instrument for assessing subjective psychological well‐being; sensitive to emotional burden (license‐free for research and clinical use; freely available, identification of source required).^89^
**PROMIS‐29** (*Patient‐Reported Outcomes Measurement Information System* – 29)	Using seven core areas, it measures various aspects of physical, psychological, and social health on a common, standardized scale enabling the comparison of patient groups. Advanced versions are available (subject to license).^38^

**TABLE 3:** Oncology‐ and HRQoL‐Specific Instruments for Assessing Quality of Life. This table summarizes relevant instruments for the detailed assessment of disease‐specific and treatment‐associated effects during survivorship. The following abbreviations are used and are not listed within the table: health‐related quality of life (HRQoL), immune checkpoint inhibitor (ICI), quality of life (QoL), and QLQ‐SURV = Quality of Life Questionnaire Survivorship.

Instrument	Description
**EORTC QLQ‐C30** (European Organisation for Research and Treatment of Cancer – *Quality of Life Questionnaire – Core* 30)	One of the most commonly used oncologic HRQoL instruments, developed in the 1980s and comprising 30 items; includes functional scales (physical, emotional, social, and others), symptom scales, and global QoL measurement (subject to approval, free of charge for academic, non‐commercial use).^39^
**EORTC *Survivorship* modules** (e. g. QLQ‐SURV)	Complementary modules for specific assessment of long‐term consequences and s*urvivorship* burdens (analogous to QLQ‐C30 subject to approval).^40^
**FACT‐M** (Functional Assessment of Cancer Therapy – Melanoma)	27 questions comprising functional scales and 16 questions of a melanoma‐specific module assessing, e. g., skin‐related symptoms, functional impairments, and fear of recurrence (subject to approval, usually free of charge for academic, non‐commercial use).^41^
**FACT‐ICM** (*Immune Checkpoint Modulators*):	A new instrument developed specifically for patients receiving ICI therapy. Instead of the melanoma module, the general questions are complemented by 25 questions on specific irAEs, e. g., skin reactions, joint problems, or gastrointestinal symptoms that will often remain relevant in the *survivorship* phase.^42^
**PRO‐CTCAE** (*Patient‐Reported Outcomes – Common Terminology Criteria for Adverse Events*)	A specific instrument for patient‐reported assessment of treatment‐related adverse events (e. g., fatigue, joint/muscle pain, skin symptoms), analogous to the CTCAE classification of adverse events by medical personnel and thus particularly suitable for complementing classical QoL instruments for questions concerning immunotherapy and *survivorship* (license‐free, freely available, identification of source required).^90^

For questions concerning *survivorship*, a combination of generic and oncological HRQoL instruments is recommended.

The QoL is usually measured as a patient‐reported outcome (PRO), either on paper or, increasingly, in electronic form (ePRO). The frequency of measurement should be guided by the therapeutic situation and follow‐up phase: prior to initiation of systemic therapy as baseline, during treatment at regular intervals (for example, every 4–12 weeks), and during the *survivorship* phase at longer intervals, for example, semi‐annually or annually, complemented by occasion‐related measurements in case of newly emerging symptoms or a change in therapy. The aim is to recognize relevant changes at an early stage and to initiate supportive measures in a targeted manner.

### Available studies on quality of life in melanoma survivors

While insights into HRQoL of melanoma survivors have increased in recent years, they remain heterogeneous with respect to populations, measurement instruments, and follow‐up duration.^35^, ^43^, ^44^, ^45^, ^46^, ^47^ The focus of the available data lies not primarily on individual treatment‐related adverse events but rather on functional, psychosocial, and everyday dimensions of QoL in the long term. Several population‐based cross‐sectional studies show that many long‐term melanoma survivors reach global HRQoL scores corresponding to those of the age‐ and gender‐matched general population. Particularly in patients after early stages or after successful primary therapy, the scores for generic quality of life often differ only marginally from reference collectives.^48^, ^49^ At the same time, differentiated subscale analyses indicate that individual functional areas (for example, physical role function, vitality, or social participation) may still be impaired even years after diagnosis, although this is not necessarily reflected in the global evaluation of quality of life.^50^, ^51^ This emphasizes the role of multidimensional instruments.

Subscale analyses show that individual functional areas, such as role function, vitality, or social participation, may be impaired even years after diagnosis.

### Psychosocial dimensions and coping with the disease

Irrespective of tumor control, many survivors report persistent psychosocial challenges. Pronounced fear of recurrence,^52^, ^53^ altered self‐perception, and disturbed body image^54^ as well as uncertainty concerning occupational and social reintegration are described particularly often.^54^, ^55^, ^56^, ^57^ Moreover, emotional burdens persist in some affected patients even with an objectively favorable prognosis.^56^, ^58^ Studies show that a relevant proportion of patients report clinically relevant anxiety symptoms or reduced emotional function, even though physical performance is largely preserved. Female gender, younger age at diagnosis, and early recurrences are associated with a lower HRQoL.^43^, ^53^, ^58^

Female gender, younger age at diagnosis, and early recurrences are associated with a lower HRQoL.

These findings illustrate that the *survivorship* phase is not to be equated automatically with psychological relief, but rather presents an independent adjustment and coping phase. Apart from the emotional dimension, functional aspects come into focus: Return to work, resumption of social roles, and subjective performance in daily life are key factors influencing QoL. Long‐term studies show that many melanoma survivors retain their basic autonomy, but report impairments in role functions, social activity, or vitality.^43^, ^59^, ^60^ Accordingly, HRQoL is increasingly understood as an expression of a functional and psychosocial adaptation and not only as the absence of medical problems; sometimes, individual areas of function and distress remain persistently impaired.^35^, ^43^ Factors resulting in an impaired HRQoL during the *survivorship* phase include advanced tumor stage at the time of initial diagnosis,^58^ relevant comorbidity,^60^ factors of psychosocial distress,^53^ and insufficient social support.^43^, ^60^ Given that this heterogeneity is often depicted incompletely by global QoL scores, the use of specific subscales and complementary psychosocial screening instruments is indicated.

## SYMPTOMS AND NEEDS OF LONG‐TERM SURVIVING MELANOMA PATIENTS

### Quality of life (QoL) in *survivorship*: Results of recent cross‐sectional studies

The clinical relevance of chronic adverse events is not primarily mortality but rather the sustained impairment of QoL. In survivorship cohorts, chronic fatigue, gastrointestinal symptoms, and musculoskeletal pain are closely associated with reduced physical and psychosocial QoL.^61^, ^62^, ^63^ Moreover, Robert et al. have shown that these symptoms may significantly impair occupational reintegration and social participation.^12^, ^64^ In this context, several *real‐world* cohorts provide detailed insight into the current care situation.^12^, ^35^, ^65^ A cross‐sectional survey of long‐term survivors (≥ 5 years after entry into stage IV) showed a high prevalence of persistent treatment‐related symptoms. Approximately 46 % of those patients who had received at least one systemic therapy reported persistent physical symptoms that they attributed to prior systemic therapy.^12^ However, surgical and radiotherapeutic interventions also cause persistent long‐term effects in a relevant proportion of patients. After surgical interventions, approximately 42 % of patients reported persistent impairments (for example, numbness, limited movement, or lymphedema). Following radiotherapy, almost 44 % of irradiated patients reported persistent symptoms, including functional limitations and somatosensory disorders; in addition, cognitive impairments, especially impaired memory and concentration, were prominent after cerebral irradiation.^12^

In a multicenter cross‐sectional study of patients with stage IV melanoma after treatment with pembrolizumab, psychological well‐being after a median follow‐up of 9.1 years did not differ significantly between the interviewed melanoma patients – including those with previous irAEs – and age‐stratified reference scores of the general population. Only in a small subgroup of those between 41 and 60 years of age, were significantly worse scores found in patients with persistent symptoms. Overall, however, survivors with irAEs during pembrolizumab therapy reported significantly lower QoL scores at follow‐up compared to patients without irAEs. A similar trend was shown in long‐term survivors with persistent symptoms, although this was not significant (p = 0.17).^35^ The role‐related functioning was assessed by means of the *Role Functioning Scale* (RFS), which captures the level of functioning in the areas of work/productivity, independent living and self‐sufficiency, as well as relationships within the immediate and extended social environment.^35^, ^66^ This was lower in long‐term survivors compared to the general population (p = 0.08).^35^

### Psycho‐oncological burden and emotional needs in reality and clinical practice

In long‐term survivors of stage IV disease, established screening instruments for assessing the psycho‐oncological burden (Distress Thermometer, Hornheide Screening Instrument) revealed that approximately one third of the respondents reached scores above the cutoff and therefore formally required psycho‐oncological support.^12^ In this context, the repeatedly observed discrepancy between objective need for psycho‐oncological support measured by screening instruments and the subjective self‐assessment of patients is notable. Significantly fewer patients reported the need for psycho‐oncological support in their self‐assessment than suggested by the screening instruments.^12^ This aspect is relevant for clinical routine, given that it may indicate a psychological barrier to the use of psychosocial help, possibly because of fear of stigmatization. There are certainly also patients who, while stressed, have sufficient social contacts/resources and, therefore, no further need for support at the time of assessment.^67^ Since these resources, as the situation of distress, are dynamic and may change over time, regular re‐screening e.g. in the framework of *survivorship* programs is indicated and necessary. Nalabothu and Johnson also emphasize the relevance of structured follow‐up programs and interdisciplinary *survivorship* concepts. Moreover, these should include regular endocrinological monitoring, neurological follow‐ups, and systematic assessment of patient‐reported symptoms.^68^

Significantly fewer patients reported the need for psycho‐oncological support in their self‐assessment than suggested by the screening instruments.

In the MESSAGE study, no correlation was found in the studied stage III cohort between therapeutic procedure or survival time and psychological distress. Accordingly, a suspected association between disease parameters and long‐term well‐being was not confirmed.^65^ In contrast, a significant association between increased psychosocial distress and persistent adverse effects of systemic therapies was identified in patients with stage IV melanoma.^12^ In stage III survivors, the mean distress was moderate, although scores were often above common cutoff values. Scores for depression and anxiety were increased compared to the general population while the global quality of life remained similar overall.^65^ This highlights a common *survivorship* paradox: Patients themselves generally rate their quality of life as good, while at the same time relevant psychological distress and disease‐related everyday stress persist.

## FOLLOW‐UP AND THERAPY PLANNING AFTER COMPLETE REMISSION UNDER IMMUNOTHERAPY

### Reality of care after complete remission under immunotherapy

**FIGURE 3:**
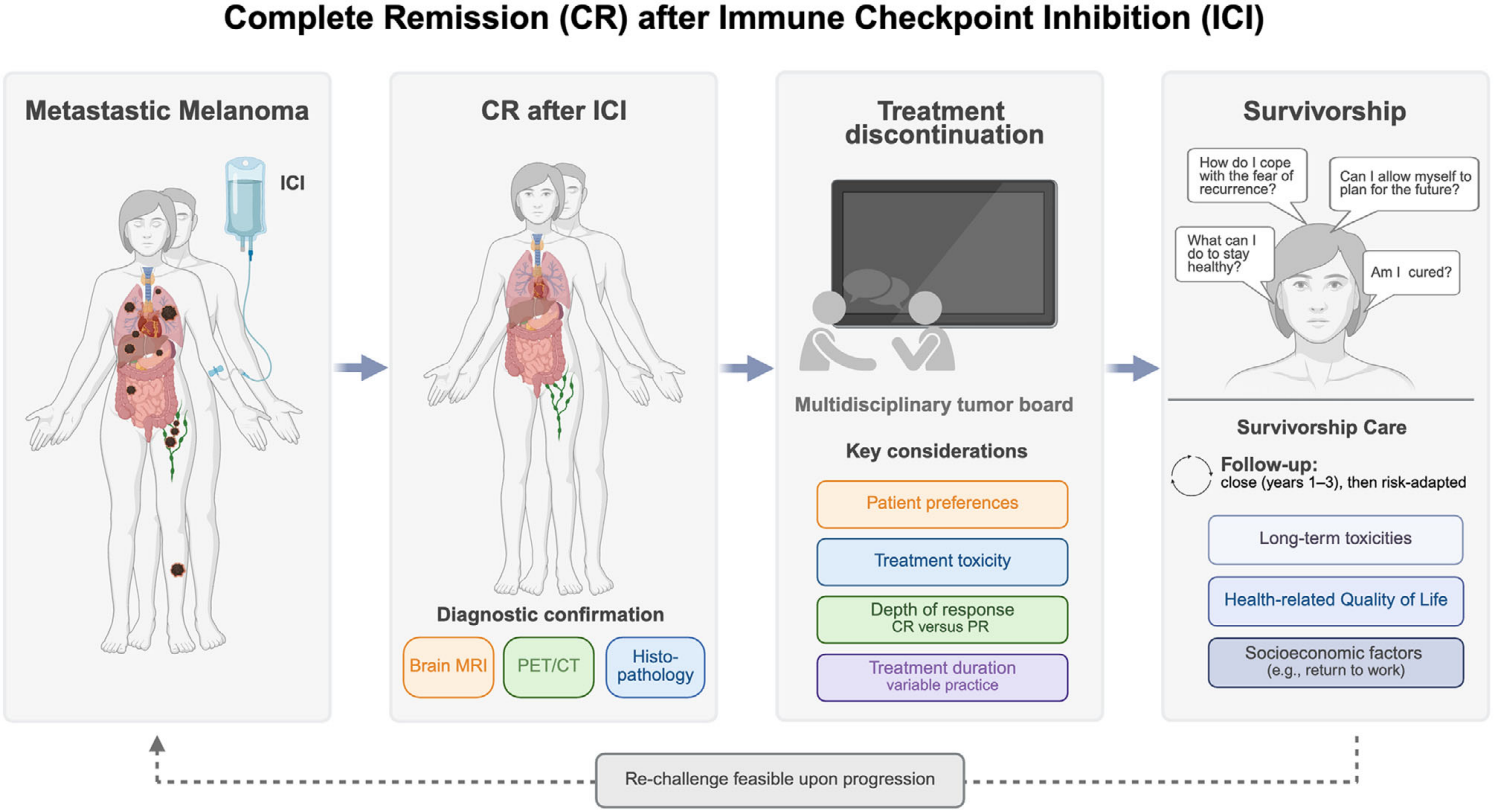
Post‐treatment surveillance and therapeutic decision‐making after complete remission (CR) achieved with immunotherapy (ICI). Schematic illustration of follow‐up strategies for patients with melanoma after achieving complete remission (CR) in the clinical routine. The figure was generated using BioRender software (BioRender.com, Toronto, Canada).

With the increasing establishment of ICIs, the treatment spectrum of metastatic melanoma has changed fundamentally. Given that a relevant proportion of patients now achieve CR under ICI treatment, the clinical questions increasingly shift from acute therapy to management of remission (Figure 3). A survey among 51 German‐speaking skin cancer centers (Germany, Switzerland, Austria) presents the most comprehensive assessment concerning the specific real‐world management after achieving CR with ICIs to date and systematically depicts the current situation of care in the German‐speaking region.^69^ In two thirds of the skin cancer centers, therapy is continued for up to six months after achieving CR under ICIs and is then terminated. Discontinuation of ICIs following CR is by no means an exception in clinical routine. At the same time, a very heterogeneous approach was found in cases of PR or SD ranging from a few months to more than two years. The decision is made predominantly in the tumor board in an interdisciplinary manner, reflecting the complex nature of this question. The high significance of patient preference is, however, notable: Treatment‐related burden, cumulative toxicities, organizational effort, and the wish to return to treatment‐free daily life, if possible, play a central role when patients express the wish for premature discontinuation of treatment. Initiation of a discussion concerning interruption of therapy without the initiative of patients was very rare.

Prior to discontinuation of ICIs, an intensive diagnostic verification of CR was conducted in the majority of centers. This almost always included cerebral imaging (MRT in 94 %) and PET/CT (in 86 % of the centers), with histological evaluation in cases where findings remained unclear. Although follow‐up after ICI discontinuation was also heterogeneous, it relied overall rather heavily on imaging, especially in the initial one to three years. In many centers, radiologic follow‐up was continued for five years or more, in some cases irrespective of individual risk factors. It became clear that this clinical practice was based on a limited evidence base and characterized by substantial variability, underscoring the urgent need for prospective data.^69^

### Need for and conducted prospective studies to date

Against the background of the current reality of care, the Safe Stop trial presented in 2025 by van der Veldt et al. at the ESMO Congress is of particular importance. It is the first completed prospective study specifically analyzing the early discontinuation of anti‐PD‐1 therapy after confirmed CR or PR. Accordingly, it differs fundamentally from previously available data that are derived predominantly from retrospective cohorts, expert surveys (for example, DeCOG/ADO), or pivotal trials with defined treatment duration, usually 24 months. It is the first study prospectively addressing a central survivorship question.

Overall, 200 patients were included, with 155 of these in stage IV. At the time of therapy discontinuation, 58 patients (29 %) had achieved CR and 142 (71 %) PR. All patients received first‐line therapy with nivolumab or pembrolizumab and had achieved CR or PR with this treatment. The median treatment duration was only 24 weeks, and the median follow‐up duration was 54 months. Within 24 months after discontinuation of ICIs, 33 of 200 patients (17 %) experienced progression. New brain metastases emerged in 9 patients (5 %), and 15 patients (8 %) died because of melanoma. In view of the stage IV collective and a median therapy duration of only six months, these event rates are remarkably low. In univariate analysis, a clear prognostic difference between CR and PR at the time of therapy discontinuation was identified. Compared to CR, PR was associated with a significantly elevated risk of progression (HR 2.21; p = 0.02).^70^ In summary, this study showed a high rate of persistent remissions after 24 months despite the early discontinuation of therapy. This strategy proved to be non‐inferior to the historical control cohort from the KEYNOTE‐006 study.^7^, ^70^, ^71^

In contrast to these data, a systematic review and meta‐analysis of 20 studies with a total of 1,832 patients with malignant melanoma published in 2025 indicated a higher recurrence risk in cases with shorter therapy duration. Based on these data, the 1‐year progression‐free survival (PFS) was 95 % in patients treated for more than two years. After treatment for 1–2 years and less than one year, the 1‐year PFS decreased to 91 % and 82 %, respectively. If patients opted for an elective interruption of treatment, the 1‐year PFS rate was significantly higher compared to the group with therapy‐related discontinuations due to adverse events (91 % vs. 79 %).^72^ For *survivorship* consultation, it is particularly relevant that progression after early discontinuation does not mean the end of therapeutic options. Resumption of ICI therapy may again achieve disease control.^73^, ^74^

Close follow‐up intervals are recommended in the initial one to three years; subsequently, they may be extended in a risk‐adapted manner. The focus should be on clinical evaluation, cerebral imaging, and consideration of QoL. In the *survivorship* situation, fatigue, long‐term endocrine toxicities, and, in particular, fear of recurrence move into the focus of care.^75^ At the same time, it is important to communicate that a recurrence does not necessarily mean a poorer prognosis. In many cases, effective disease control can be achieved again.

For *survivorship* consultation, it is particularly relevant that progression after early discontinuation does not mean the end of therapeutic options. Many patients can be treated successfully with ICIs again, in some cases in combination with local surgical or stereotactic interventions.

## *SURVIVORSHIP* CARE PROGRAMS: OUTLOOK AND STATUS OF CURRENT RESEARCH

The project “Living with melanoma” (LeMela) sponsored by the German Cancer Aid is concerned with the development and testing of a *survivorship care* program for patients with metastatic melanoma during and after therapy. In a scoping review conducted in the framework of the LeMela program, seven studies (nine articles) met the inclusion criteria and were included in the analysis. Six *survivorship care* concepts and associated components could be identified. These include oncological follow‐up (related to physical functions),^76^, ^77^ psychosocial follow‐up,^76^, ^77^, ^78^ monitoring for recurrences and new cancer diseases,^77^, ^79^, ^80^ resources and coordination of care,^76^, ^77^ information and empowerment,^76^, ^77^, ^81^ and so‐called multimodal approaches (Table 4).^82^, ^83^, ^84^

**TABLE 4:** Identified Survivorship Care Concepts. Overview of survivorship care concepts for melanoma patients identified within the scope of the “Living with Melanoma” (LeMela) scoping review project. The scoping review was conducted according to the Joanna Briggs Institute methodology, and the search strategy was developed using the PCC framework (Population, Concept, Context).

Survivorship care concepts	Description
**Oncological follow‐up (with respect to physical functions)** ^76^, ^77^	Screening and management of acute and chronic, immune‐related adverse events, consideration of impact on fertility, screening for endocrinopathies, assessment of lymphedemas, screening for fatigue, and consideration of referral to palliative medicine.
**Psychosocial follow‐up** ^76^, ^77^, ^78^	Screening for anxiety and depression, mapping of more local access to psychologists and psychiatrists, assessment of the situation concerning social work, and screening for financial burden, and spiritually oriented meditation.
**Monitoring for recurrences and new cancer diseases** ^77^, ^79^, ^80^	Skin and lymph node examinations, routine blood tests and imaging as well as digital, tablet‐based intervention to support complete self‐examination.
**Resources and coordination of care** ^76^, ^77^	*Survivorship care* plan, multidisciplinary cooperation and designated contact persons.
**Information and *empowerment*** ^76^, ^77^, ^81^	Personalized information/consultation, e. g., about risk factors and provision of information about reliable sources, social support by telephone guided by peers.
**Multimodal approach** ^82^, ^83^, ^84^	*Model of nurse‐led, telehealth‐delivered survivorship care* (MELCARE) and *Melanoma Care Study*.

The identified *survivorship care* concepts are often characterized by a multimodal approach. Three of the seven studies involved patients with stage III–IV melanoma.^76^, ^78^, ^82^ Two other studies provided no detailed information about the target group,^77^, ^81^ while two studies focused on patients with stage 0–IIc melanoma.^79^, ^80^, ^83^, ^84^ Implementation of the identified *survivorship care* concepts requires significant investments in human and technological resources.^78^, ^79^, ^80^, ^82^, ^84^ Nevertheless, individual concepts, such as psychosocial follow‐up and monitoring for recurrences and new cancer diseases, seem to reduce fears and depressive symptoms^83^ and improve the QoL.^79^ There is a gap in the empirical research with respect to health literacy in the context of *survivorship care*. Tailored information and demand‐driven interventions that also consider individual risks of potential negative medical and psychosocial consequences are key components in the implementation of *survivorship care* plans for cancer patients in general.^85^ In view of the wealth of available health information and the increasing use of digital health applications, supporting patient health literacy should be given high priority.^85^ Moreover, health economic analyses are lacking. Additional studies are required to analyze the efficacy of *survivorship care* concepts. The results will support the development of a patient‐centered *survivorship care* program for patients with melanoma.

## REHABILITATION

**FIGURE 4:**
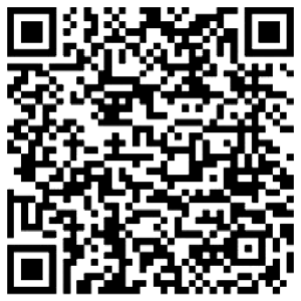
QR Code, Das Rehaportal.

The S3 guideline on melanoma emphasizes that the follow‐up should consider functional, psychosocial, and social consequences of the disease in addition to tumor control. In this context, oncological rehabilitation is mentioned as a component of a biopsychosocial care concept, especially for the treatment of fatigue, psychological distress, and impairments of participation in daily life and gainful employment.^21^ Generally, all patients in Germany have a legal claim to medical rehabilitation with the aim of re‐establishing or securing participation in social and occupational life (§ 40 SGB V, § 9 SGB VI, § 31 SGB XI). According to the German Social Code (*Sozialgesetzbuch*, SGB), patients have the right to freely choose a suitable rehabilitation center themselves (§ 8 SGB IX). (Dermato)‐oncological rehabilitation can be requested following curative‐intent therapy, during maintenance therapy, and in the palliative situation. An overview of potentially suitable rehabilitation centers for the disease code C43 (malignant melanoma of the skin) is available via the following online portal (see QR code, Figure 4).

Prerequisites are sufficient motivation, resilience, and independence of patients (rehabilitation capability) as well as an identified need for rehabilitation in the sense of health‐related impairments with a realistic prospect of improvement. If functional impairments persist, there is an opportunity to apply for further rehabilitation measures (https://www.deutsche‐rentenversicherung.de/DRV/DE/Reha/Reha‐Antragstellung/reha‐antragstellung_node.html; www.reha‐hilft‐krebspatienten.de). Although specific randomized studies on rehabilitation in melanoma survivors are limited to date, the benefit of oncological rehabilitation is well established. Current meta‐analyses show that exercise‐based and multimodal rehabilitation programs result in significant improvements in fatigue, physical performance, HRQoL, anxiety, and depression in cancer patients. These effects involve central areas of concern in melanoma *survivorship* and support the transferability of rehabilitation concepts to this patient group.^86^, ^87^, ^88^ Against this background, oncological rehabilitation should be integrated into follow‐up planning for melanoma patients at an early stage. In this context, dermatologists play a key role, given that they provide long‐term support to patients in the framework of follow‐up programs.

Generally, all patients in Germany have a legal claim to medical rehabilitation with the aim to re‐establish or secure the participation in social and occupational life (§ 40 SGB V, § 9 SGB VI, § 31 SGB XI).

## SUMMARY AND OUTLOOK

For decades, the prognosis for metastatic melanoma was poor; however, the introduction of ICIs has led to a dramatic improvement in overall survival. Long‐term survivors are now attending our clinics and presenting clinicians with new challenges. In particular, chronic immune‐mediated toxicities, the return to “normal life,” and occupational reintegration pose challenges for both patients and healthcare providers. Against the background of a potentially achievable long‐term survival despite previous unresectable metastases, this aspect must be considered in therapy planning from the start. Given the improved treatment options, patients are now generally able to experience long‐term toxicities, for example, after radiation. Rehabilitation measures and *survivorship* programs are therefore of great importance, and new areas of research are emerging. A “*Survivorship*” committee was established within the ADO, and a new professorship in “*Cancer Survivorship*” was created in Leipzig in 2025 (as of 02/2026). Nevertheless, nationwide *survivorship care* programs, such as those in place for breast cancer, need to be established for melanoma. To this end, implementation of telemedicine consultations or app‐based follow‐up programs may be helpful. Interdisciplinary cooperation is certainly the key to providing optimal care for long‐term survivors.

## CONFLICT OF INTEREST STATEMENT

MR has received a grant in the framework of the Junior Clinician Scientists Program of Tuebingen University (application number 523‐0‐0) and travel allowances from Almirall Hermal, Galderma, and Pierre Fabre, none of which were related to the submitted work.

SDB has received an honorarium for a lecture from Medea GmbH that was not related to the submitted work.

CLB has received a grant in the framework of the Mildred Scheel Nachwuchszentrum (MSNZ) of the University Medical Center Hamburg‐Eppendorf (UKE) financed by the German Cancer Aid that is not related to the submitted work.

EL reports advisory activity or has received honoraria/travel costs from: Bristol‐Myers Squibb, Merck Sharp & Dohme, Kyowa Kirin, Novartis, Pierre Fabre, Regeneron, Sanofi, Sunpharma, and Takeda.

MS reports advisory activity or has received honoraria/travel costs from: Bristol‐Myers Squibb, Merck Sharp & Dohme, Immunocore, Novartis, Pierre Fabre, Regeneron, and Sunpharma, all unrelated to the submitted work.

MVH has received honoraria for advisory activity from Novartis, Immunocore, BMS, MSD, Sanofi, Almirall, Pierre Fabre, Infectopharm, and Galderma, all unrelated to the submitted work.

GS has worked as advisor for Novartis, unrelated to the submitted work.

FM has worked as advisor and/or has received honoraria from Novartis, Bristol‐Myers Squibb, Merck Sharp & Dohme, Pierre Fabre, Sanofi Genzyme, and Sun Pharma as well as travel allowances from Novartis, Sun Pharma, Pierre Fabre, and Merck Sharp & Dohme, unrelated to the submitted work.

LZ reports advisory activity or has received honoraria/travel costs from the following companies: Bristol‐Myers Squibb, Merck Sharp & Dohme, Novartis, Pierre Fabre, Regeneron, Sanofi, and Sunpharma.

AF has worked as advisor for Novartis, MSD, BMS, Pierre‐Fabre, and Immunocore; received travel allowances from Novartis, BMS, and Pierre‐Fabre as well as honoraria for lectures from Novartis, BMS, and MSD; she reports institutional grants from the BMS Stiftung Immunonkologie that are not related to the submitted work.

The other authors declare that they have no conflicts of interest.

## [CME QUESTIONS – LERNERFOLGSKONTROLLE]

1. Welche Aussage zur Definition des „*Cancer Survivor*“ ist korrekt?
  - Als *Cancer Survivor* gelten ausschließlich Patienten, die mindestens fünf Jahre nach Krebsdiagnose überlebt haben.
  - Ein *Cancer Survivor* ist jeder Mensch ab Abschluss der Krebstherapie bis zum Lebensende.
  - Der Begriff *Cancer Survivor* beschreibt nur Patienten ohne aktive Tumorerkrankung.
  - *Cancer Survivor* sind ausschließlich Patienten mit kompletter Remission nach einer Krebserkrankung.
  - Ein *Cancer Survivor* ist jeder Mensch vom Zeitpunkt der Krebsdiagnose bis zum Lebensende – unabhängig von Krankheitsstadium oder Verlauf.
2. Wie definiert die *Society for Immunotherapy of Cancer (SITC)* ein chronisches immunvermitteltes unerwünschtes Ereignis (chronisches irAE)?
  - Nebenwirkungen, die 3 Monate nach Absetzen der ICI‐Therapie persistieren
  - Nebenwirkungen, die während der laufenden Immuntherapie auftreten
  - Nebenwirkungen, die länger als 6 Monate nach Krebsdiagnose bestehen
  - Nebenwirkungen, die ausschließlich eine immunsuppressive Therapie erfordern
  - Nebenwirkungen, die erst mehr als 12 Monate nach Therapieende auftreten
3. Welche Aussage zu endokrinen Langzeittoxizitäten unter Immuncheckpoint‐Inhibitoren beim Melanom ist korrekt?
  - Endokrine irAEs treten selten auf und sind meist vollständig reversibel.
  - Endokrine Nebenwirkungen entstehen ausschließlich spät nach Therapieende.
  - Hypothyreose und Hypophysitis sind häufig irreversibel und erfordern meist eine lebenslange Hormonsubstitution.
  - Endokrine irAEs verursachen keine langfristigen funktionellen Einschränkungen.
  - Eine Verlaufskontrolle ist nach Therapieende nicht erforderlich.
4. Welche Aussage zu Fertilität und systemischer Melanomtherapie trifft zu?
  - Robuste klinische Daten zeigen, dass ICI‐Therapien die Fertilität dauerhaft stark beeinträchtigen.
  - BRAF‐ und MEK‐Inhibitoren sind sicher für die Fertilität und haben keine embryo‐ oder fetotoxischen Effekte.
  - Männer sind von Fertilitätsproblemen unter zielgerichteter Therapie grundsätzlich nicht betroffen.
  - Endokrine irAEs können indirekt die Fertilität beeinträchtigen und erfordern bei Kinderwunsch endokrinologische Mitbetreuung.
  - Eine Fertilitätsberatung vor Beginn der Therapie ist nur bei Frauen erforderlich.
5. Welche Empfehlung zum Fertilitätserhalt vor systemischer Melanomtherapie ist zutreffend?
  - Kryokonservierung von Ovarialgewebe ist bei Männern die Methode der Wahl.
  - Eine standardisierte Fertilitätsberatung sollte bei allen Patienten im reproduktiven Alter vor Therapiebeginn erfolgen.
  - GnRH‐Agonisten schützen nur Männer vor Chemotherapie‐induzierten Fertilitätsschäden.
  - Fertilitätsmaßnahmen sind nur bei metastasiertem Melanom indiziert.
  - Eine Kryokonservierung ist bei Frauen während laufender systemischer Therapie kontraindiziert und nie empfohlen.
6. Welche Aussage zur Messung der gesundheitsbezogenen Lebensqualität (HRQoL) bei Melanom‐Überlebenden ist korrekt?
  - HRQoL‐Instrumente erfassen ausschließlich körperliche Symptome und werden daher nur in der Akutphase eingesetzt.
  - Generische Instrumente sind auf Melanompatienten zugeschnitten und ersetzen onkologische HRQoL‐Fragebögen.
  - Eine Kombination aus generischen und onkologischen HRQoL‐Instrumenten ermöglicht sowohl Vergleichbarkeit mit der Allgemeinbevölkerung als auch die Erfassung krankheitsspezifischer Belastungen.
  - *Patient‐Reported Outcome*‐Instrumente (PROMs) sollten ausschließlich papierbasiert erhoben werden, um technische Barrieren zu vermeiden.
  - Subskalenanalysen sind nicht notwendig, da globale QoL‐Werte alle Dimensionen abbilden.
7. Welche Aussage zum Langzeitverlauf der Lebensqualität bei Melanom‐Überlebenden trifft am besten zu?
  - Nach erfolgreicher Primärtherapie erreichen nach einiger Zeit alle Patienten wieder die QoL‐Werte der Allgemeinbevölkerung.
  - Einschränkungen in Rollenfunktion, sozialer Teilhabe oder Vitalität können langfristig bestehen, selbst wenn die körperliche Autonomie erhalten bleibt.
  - Psychosoziale Belastungen wie Angst vor Rezidiv oder Veränderungen im Körperbild treten nur während der Therapie auf.
  - Geschlecht oder Alter bei Diagnose haben keinen Einfluss auf die QoL.
  - Globale QoL‐Scores erfassen zuverlässig alle funktionellen und psychosozialen Einschränkungen.
8. Welche Aussage zur Nachsorge und Therapieplanung nach kompletter Remission (CR) unter Immuntherapie beim metastasierten Melanom ist zutreffend?
  - Ein Progress nach frühem Absetzen der ICI‐Therapie bedeutet zwingend eine schlechte Prognose.
  - Das Absetzen der ICI nach CR ist in der klinischen Praxis äußerst selten.
  - Nachsorgeintervalle sind in allen Fällen risikoadaptiert und standardisiert.
  - Patienten mit PR (partieller Remission) zum Zeitpunkt des Therapieabbruchs haben ein höheres Progressionsrisiko als Patienten mit CR.
  - Eine Wiederaufnahme der ICI‐Therapie nach Progress ist nicht möglich.
9. Welche Komponenten sind typische Bestandteile von *Survivorship‐Care*‐Programmen für Melanompatienten?
  - Nur onkologische Nachsorge der körperlichen Funktionen
  - Survivorship‐Care beschränkt sich auf Patienten im Stadium I
  - Ausschließlich digitale Gesundheitsanwendungen
  - Nur medizinische Diagnostik ohne psychosoziale Aspekte
  - Psychosoziale Nachsorge, Überwachung auf Rezidive, Ressourcenkoordination sowie Information/*Empowerment*.
10. Welches der folgenden Statements zur onkologischen Rehabilitation von Melanompatienten in Deutschland ist richtig?
  - Alle Patienten haben grundsätzlich einen gesetzlichen Anspruch auf medizinische Rehabilitation, um soziale und berufliche Teilhabe wiederherzustellen oder zu sichern.
  - Eine Reha kann nur während der kurativen Therapie beantragt werden.
  - Medizinische Rehabilitation ist nur Patienten mit metastasiertem Melanom vorbehalten.
  - Die Wahl der Rehabilitationseinrichtung ist ausschließlich durch den behandelnden Arzt festgelegt.
  - Onkologische Rehabilitation zeigt bei Melanompatienten keinen nachgewiesenen Nutzen für Fatigue, körperliche Leistungsfähigkeit oder Lebensqualität.

Liebe Leserinnen und Leser, der Einsendeschluss an die DDA für diese Ausgabe ist der 30. November 2026.

Die richtige Lösung zum Thema Palmoplantare Pustulose: Entstehung, Differentialdiagnose und Therapie in Heft 4/2026 ist: 1c, 2b, 3d, 4c, 5a, 6b, 7b, 8b, 9b, 10c

Bitte verwenden Sie für Ihre Einsendung das aktuelle Formblatt auf der folgenden Seite oder aber geben Sie Ihre Lösung online unter http://jddg.akademie-dda. de ein.
